# The prescription of opoides analgesics: what preventive measures are taken by doctors to avoid their problematic use?

**DOI:** 10.1192/j.eurpsy.2023.1383

**Published:** 2023-07-19

**Authors:** L. Tbatou, B. Yassmine, E. O. Fatima

**Affiliations:** psychiatric emergency, Hospital ArRazi, salé, Morocco

## Abstract

**Introduction:**

An opioid analgesic exposes you to a risk of problematic use, due to the terrain (vulnerability), its emotional state, the presence of pain and the environment and thus the opioid itself. The risk of developing an opioid use disorder when taking prescription opioids is approximately 3% (over 2 years),

**Objectives:**

Recognize the method of prescription of opioids, as well as the elements to be sought by doctors during the interrogation and after each prescription of opioids

**Methods:**

a descriptive and analytical study, based on a hetero-questionnaire completed by doctors, numbering 120 doctors who were consulted over a period of one year. the study was carried out via Google Forms.

**Results:**

75% of doctors are general practitioners. 90% of our doctors have a period of practice less than 10 years, and the lower half of 4 years, Almost all doctors prescribe opioid analgesics for pain of severe intensity, often of rheumatic origin 50%, neoplastic, post-operative or for a migraine or neuralgia. 9% of physicians think opioid painkillers have a high risk of addiction, almost 20% think opioid painkillers do not even have a risk of addiction, and the majority think the risk is low to moderatedoctors limit the duration of prescription 80%, sensitize their patients in 75%, half prescribe a low dose but the majority abruptly stop treatment. 70% of doctors limit the duration of prescription in less than 02 weeks, but 15% do not limit not prescribing opioids

**Conclusions:**

The dose-efficacy-tolerance relationship is very variable from one patient to another, it is important to adapt the dosage gradually according to the intensity of the pain with monitoring of possible tolerance. Stopping should be done gradually to avoid a withdrawal syndromeThe prescription must systematically be accompanied by information to the patient about the treatment and its discontinuation, and monitoring of these risks even when it is prescribed in compliance with the conditions of the marketing authorization.

**Disclosure of Interest:**

None Declared

